# Return to Play After Achilles Tendon Rupture: Comparing Operative and Nonoperative Approaches in Athletes

**DOI:** 10.7759/cureus.96783

**Published:** 2025-11-13

**Authors:** Laith Fada, Alexander Mazzorana, Zachary A Grand, Greg Jacobs

**Affiliations:** 1 Medicine, Alabama College of Osteopathic Medicine, Dothan, USA; 2 Medicine, Florida International University Herbert Wertheim College of Medicine, Miami, USA; 3 Emergency Medicine, Alabama College of Osteopathic Medicine, Dothan, USA

**Keywords:** achilles tendon rupture, athletes, non-operative rehabilitation, operative management, return to play

## Abstract

Achilles tendon rupture is a debilitating injury with significant implications for athletic performance and career longevity. Historically, operative repair was considered the gold standard, but advances in early functional rehabilitation have challenged this paradigm, with non-operative management demonstrating comparable rerupture and functional outcomes. This narrative review synthesizes evidence comparing operative and nonoperative strategies in athletes, emphasizing rehabilitation, return-to-play (RTP) outcomes, and long-term sequelae. Operative repair offers marginal advantages in preserving tendon length and explosive power but carries surgical risks such as adhesions and wound complications. Nonoperative management, utilizing modern functional protocols, achieves comparable rerupture rates and patient satisfaction while minimizing operative morbidity. It appears that the quality of rehabilitation and early management offers promising results in RTP, comparable to those of surgery.

## Introduction and background

The Achilles tendon is the largest and strongest tendon in the human body, playing a central role in explosive activities such as sprinting, jumping, and cutting [1]. Rupture typically occurs during rapid acceleration, deceleration, or jumping maneuvers and carries profound implications for athletic performance and career longevity [2]. For athletes, the stakes are particularly high, as deficits in push-off strength, endurance, or confidence may compromise return to play (RTP) and shorten competitive careers [2].

Historically, operative repair was considered the gold standard, thought to reduce rerupture risk and better restore tendon length and mechanics [3]. Young, athletic patients underwent surgery, while older or less active patients were often managed conservatively [4]. In recent years, however, evidence has challenged this dogma. With the introduction of early functional rehabilitation and protected weight-bearing, nonoperative treatment has shown outcomes comparable to those of surgery in many populations [5].

To refine patient selection, imaging-based algorithms such as the Copenhagen Achilles Rupture Treatment Algorithm (CARTA) have been proposed [6]. The CARTA integrates ultrasound assessment of tendon end apposition and elongation to guide treatment decisions, moving beyond age or activity level alone [6]. At the same time, surgical options have diversified, from open repairs to minimally invasive techniques, while rehabilitation strategies emphasizing early mobilization and dynamic bracing have become increasingly central [6]. This review synthesizes current evidence comparing operative and nonoperative management of Achilles tendon ruptures in athletes, with particular emphasis on rehabilitation strategies, RTP outcomes, and long-term sequelae.

## Review

Epidemiology

Achilles tendon rupture accounts for up to 20% of all major tendon injuries, with an annual incidence estimated at five to 10 per 100,000 individuals in the general population [7]. Rates are significantly higher in athletes, particularly males between the ages of 30 and 50 who participate in sports requiring explosive acceleration, deceleration, or jumping, such as basketball, soccer, and racquet sports.

Elite athletes face an even greater burden, as rupture often occurs during peak competition years. While overall RTP rates are encouraging, performance decrements and shortened careers are reported across multiple professional leagues [8]. The rising popularity of recreational sports among middle-aged adults has also contributed to an increase in rupture incidence in non-elite populations [9].

Pathophysiology

The Achilles tendon is subjected to some of the highest mechanical loads in the body, particularly during explosive athletic movements [10]. Ruptures most commonly occur 2 cm to 6 cm proximal to the calcaneal insertion in an area known as the 'watershed zone' [11]. Acute injury typically results from a sudden eccentric load on a contracted tendon, such as during sprint take-off or landing from a jump [11].

Achilles tendon degeneration involves mechanical overload, microvascular compromise, and molecular dysregulation. Histologic studies demonstrate that the areas most likely to rupture already have microscopic degenerative changes. These include disorganized and weakened collagen fibers, accumulation of mucoid material within the tendon, and increased apoptosis of tenocytes. Together, these changes reduce the tendon’s strength and ability to handle high loads, making it more vulnerable to rupture [12]. Inflammatory mediators, including IL-1β and TNF-α, contribute to extracellular matrix breakdown by stimulating matrix metalloproteinases and impairing normal collagen synthesis, while reduced type I collagen and increased type III collagen weaken the tendon’s structural integrity [13]. 

Metabolic factors, including obesity, diabetes, and hyperlipidemia, further compromise tendon health by promoting oxidative stress, advanced glycation end-product accumulation, and reduced perfusion, accelerating degenerative change even in athletic individuals [14]. While intrinsic healing often results in tendon elongation and reduced plantarflexion power, emerging biologic therapies such as platelet-rich plasma (PRP), growth-factor-based therapy, and stem-cell strategies aim to enhance matrix remodeling and tendon regeneration when paired with structured rehabilitation programs [15]. Together, these biomechanical and biochemical mechanisms highlight the need for individualized treatment strategies combining mechanical loading optimization with biologic modulation.

Current treatment algorithms

**Figure 1:**
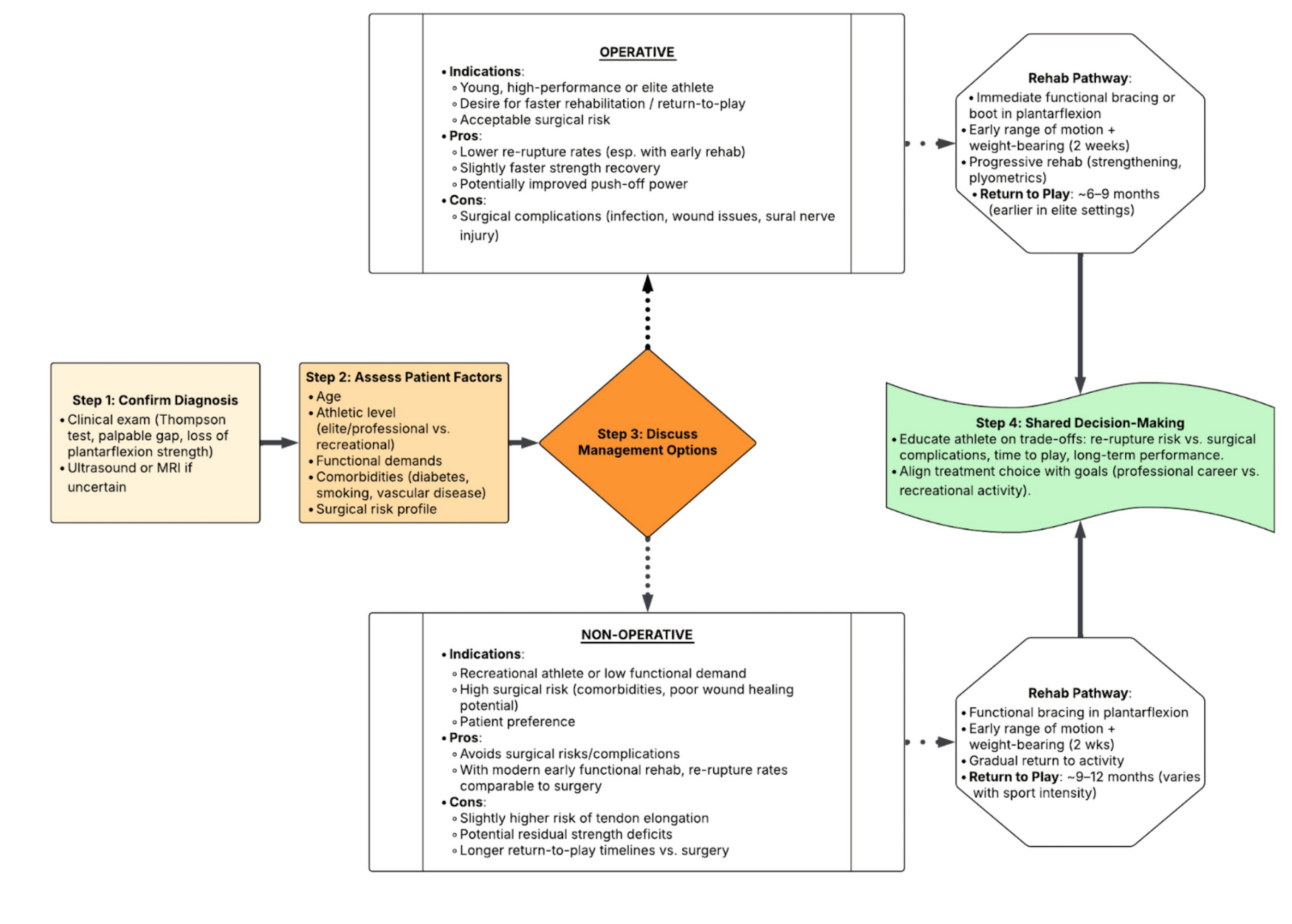
Clinical decision pathway for Achilles tendon rupture management The flow diagram outlines operative and nonoperative treatment options, incorporating modern functional rehabilitation and individualized algorithms such as the CARTA. This diagram was created by the authors. CARTA: Copenhagen Achilles Rupture Treatment Algorithm

As demonstrated in Figure 1, the operative versus nonoperative debate has shifted away from reflexive surgical selection toward imaging-guided, individualized management frameworks [5]. The CARTA represents one such model, which uses ultrasound measurements to objectively check how well the torn tendon ends line up to guide treatment choice rather than relying solely on athletic status or age [6]. Early prospective data suggest that CARTA-guided decision-making achieves rerupture rates comparable to surgical cohorts when nonoperative care is selected appropriately, with reported sensitivity of 84% and specificity of 91% for identifying patients likely to succeed without surgery, along with improved functional scores in correctly stratified patients [6]. However, validation in elite athletic populations remains limited, and most evidence derives from mixed recreational cohorts, making generalizability to high-performance settings incomplete.

Figure 1 illustrates how decision-making has evolved toward algorithmic, objective evaluation, emphasizing tendon morphology and healing potential rather than historical assumptions. This reflects a broader trend: some centers trial functional rehabilitation first and reserve surgery for early non-responders [16], whereas many elite sports environments continue to favor primary surgical repair, citing performance demands, explosiveness requirements, and psychological assurance associated with operative stabilization [1]. Taken together, these data reinforce the notion that RTP outcomes depend not only on surgical choice but also on appropriate patient selection, quality of rehabilitation, and prevention of tendon elongation, the primary determinant of functional deficit in athletes [17].

Operative management

Operative repair of Achilles tendon rupture has traditionally been favored in athletes due to evidence that it better restores tendon structural integrity, length, and tension, thereby minimizing rerupture rates and facilitating a faster RTP [5]. Early studies consistently demonstrated rerupture rates of <5% in surgically treated cohorts, nearly half the rate observed in patients managed conservatively with prolonged casting [5]. In addition, multiple reports suggest that surgery provides a more reliable recovery of calf strength and endurance, metrics that are critical for high-level athletic performance [16,17].

Over time, surgical techniques have evolved to retain these functional advantages while reducing the risks associated with open procedures [16]. Comparative meta-analyses demonstrate that minimally invasive methods, including percutaneous repairs with devices such as the Achillon and Tenolig®, have demonstrated comparable biomechanical stability to open repair while decreasing wound morbidity [18]. Some authors have reported accelerated timelines for rehabilitation initiation and earlier progression to weight-bearing and running with these minimally invasive techniques, an especially relevant factor for athletes facing pressure to return to competition [17].

Despite these advancements, surgery carries unique risks. Beyond rerupture, postoperative complications include wound breakdown, superficial infection, sural nerve neuritis, and symptomatic scar formation [19]. Among these, adhesion formation is particularly impactful, reported in up to 6% of athletes after repair [20,21]. Adhesions impair tendon glide during repetitive loading, resulting in inefficient force transfer from the calf musculature to the foot. Athletes may report diminished push-off strength, delayed explosiveness, and premature fatigue, occasionally requiring adhesiolysis for relief [19,20]. Importantly, most complications, though infrequent, carry performance-limiting consequences in high-demand populations. This underscores that operative management can also be accompanied by limiting side effects.

Long-term outcomes following operative repair are generally favorable, with RTP rates reported between 70% and 90% [16]. However, even among athletes who return, performance often declines, and lingering deficits in plantar-flexion strength, rate of force development, and endurance have been documented despite successful repair [17]. Psychological readiness to return has also emerged as a significant barrier in elite cohorts, with fear of reinjury, reduced confidence in explosive push-off, and altered kinematics contributing to delayed or incomplete RTP [17]. Collectively, current evidence suggests that while surgery remains an important option, its advantage is not absolute, and outcomes are heavily dependent on surgical technique, individualized rehabilitation, and integrated psychological support rather than the procedure itself.

Nonoperative management

Nonoperative treatment was historically regarded as inferior, owing to higher rerupture rates and disappointing functional outcomes associated with cast immobilization [5]. However, the introduction of early functional rehabilitation has dramatically altered this perspective [22]. Contemporary nonoperative protocols emphasize immediate plantarflexed immobilization in a functional boot, followed by graduated dorsiflexion and controlled weight bearing within the first weeks after injury [23]. Mechanical loading during early healing promotes collagen alignment, reduces tendon elongation, and minimizes muscle atrophy and neuromuscular decline compared with prolonged casting [24].

While accelerated rehabilitation has narrowed the outcome gap, its effectiveness relies heavily on adherence and appropriate early loading. Poor compliance, premature dorsiflexion, or insufficient tendon end apposition may increase the risk of tendon elongation and rerupture, variables that explain discrepancies across trials [5]. Willits et al. demonstrated equivalent rerupture rates at two years (2.8% nonoperative vs. 2.6% operative) using a strictly supervised early-mobility protocol, whereas Myhrvold et al. reported higher rerupture risk with nonoperative care when rehabilitation was less tightly standardized (6.2% vs. 0.6%) [5,16]. These findings underscore that the success of nonoperative treatment is highly dependent on strict adherence to early functional rehabilitation protocols. Rerupture rates and functional outcomes vary significantly based on early loading criteria, tendon end-apposition, and patient compliance, with studies demonstrating excellent results only when rehabilitation is supervised, standardized, and initiated in a controlled manner.

A significant limitation of current evidence is the lack of trials in elite athletes. No study to date has directly compared rerupture rates or RTP outcomes between operative and nonoperative approaches in professional sport populations, leaving decision-making in athletes partly extrapolated from general population data [25]. Although nonoperative care avoids wound complications, adhesions, and sural nerve injury associated with surgery, concerns persist regarding strength, explosive power, and tendon elongation, particularly in sports demanding maximal push-off [26]. Still, multiple contemporary studies report no meaningful difference in long-term function or RTP when high-quality functional rehabilitation is applied [26].

From an athlete's standpoint, nonoperative treatment offers a viable pathway that eliminates surgical morbidity while still enabling competitive return. The RTP rates increasingly mirror those of surgical cohorts, though timelines may be modestly delayed in some series [5,16]. Psychological factors remain critical: many athletes perceive surgery as the 'stronger' option, associating it with greater structural security [27]. However, structured nonoperative programs that incorporate graded loading, objective strength benchmarks, and psychological readiness training can mitigate these concerns and support return to high-level sport [27].

Recent interest in biological augmentation, including platelet-rich plasma (PRP) and growth-factor-mediated therapies, may further enhance tendon healing in nonoperative protocols [28]. Although early studies suggest improved collagen organization and reduced tendon elongation, evidence remains preliminary, and athlete-specific data are limited, representing an important future research direction. Table 1 compares operative and nonoperative management.

**Table 1:** Comparison between operative and non-operative management of Achilles tendon rupture RTP: Return to play

Domain	Operative management	Nonoperative management (functional rehab)
Rerupture rates	Typically <5% with modern techniques [2,9]	Historically higher, but with early functional rehab, it can approach surgical rates [9,2]
Strength and endurance	Better preservation of calf strength and push-off power, though subtle deficits may persist [3]	Slightly greater deficits in endurance and explosiveness, but often modest [4]
Tendon length	More reliably restores tendon length and tension [5]	Greater risk of tendon elongation, which may reduce strength [29]
RTP rates	70% to 90% overall; often faster with minimally invasive repair [8,13]	Increasingly comparable RTP, though timelines may be modestly delayed [7]
Complications	Risks include adhesions, wound issues, and sural nerve injury [4,6]	Avoids surgical complications; lower overall risk [18]
Psychological factors	Viewed as a 'stronger' option, instilling confidence in tendon integrity [23]	Sometimes perceived as less robust; structured rehab and support improve acceptance [23]

Rehabilitation strategies

Rehabilitation has become the cornerstone of successful management in both operative and nonoperative care, and its quality often dictates the eventual RTP [29]. Early mobilization is now standard, with evidence showing that controlled weight bearing and progressive ankle motion reduce rerupture risk and improve functional recovery compared to prolonged casting [23,24]. In athletes, this principle is particularly important; immobilization not only delays tendon healing but also leads to rapid muscle atrophy and neuromuscular deconditioning that jeopardize performance on return [26].

Functional bracing is widely used to facilitate this process. Boots with heel wedges maintain plantarflexion initially, protecting the repair or healing tendon while permitting gradual dorsiflexion adjustments. This gradual reduction of plantarflexion over six to eight weeks allows for progressive tendon loading and promotes remodeling in alignment with sport-specific stresses [23]. Afterwards, emphasis shifts to calf strengthening, with eccentric loading exercises proving most effective in enhancing tendon stiffness and restoring the explosive capacity required for sprinting and jumping [23].

Neuromuscular retraining is equally vital. Plyometric drills, balance training, and sport-specific movement patterns are progressively introduced to bridge the gap between clinical recovery and competitive readiness [23]. These interventions not only restore physical performance but also reduce the risk of compensatory movement strategies that may predispose athletes to secondary injuries. Psychological rehabilitation is an increasingly recognized but often underappreciated component. Fear of rerupture can limit effort in training and delay return to competition, even in the presence of objective healing. Structured programs that integrate graded exposure to sport demands, mental skills training, and clear communication regarding risk can significantly improve athlete confidence [30].

Return to play

The most meaningful outcome for athletes sustaining an Achilles tendon rupture is the ability to return to sport, both in terms of timing and level of performance achieved. Operative repair was historically assumed to confer superior results, based on early studies reporting lower rerupture rates and faster recovery of plantarflexion strength [16]. More recent randomized controlled trials and meta-analyses have challenged this assumption, showing that RTP is more strongly determined by the quality of rehabilitation than by surgical vs. nonoperative status alone [23].

Surgical cohorts report RTP rates of 70% to 90%, with some elite athletes resuming competition as early as six to seven months postoperatively [5]. Despite these encouraging numbers, many fail to return to their pre-injury performance level. Persistent deficits in plantarflexion strength and heel-rise endurance are frequently documented, even after surgical repair [22]. Operative repair may mitigate tendon elongation and better preserve push-off strength, which is particularly valuable in sports demanding maximal explosiveness. Nonetheless, isokinetic testing consistently reveals measurable asymmetries compared with the contralateral limb [16].

Nonoperative management with early functional rehabilitation has produced increasingly comparable outcomes. Meta-analyses report RTP rates of 65% to 85%, with most athletes returning between eight and 12 months after injury [5,16]. When early mobilization and progressive loading are employed, differences in timing compared with surgery are minimal, and rerupture rates approximate those of operative cohorts [17]. Some studies report slightly greater strength and endurance deficits in nonoperatively managed patients, particularly in single-leg heel-rise performance, calf circumference, and isokinetic plantarflexion torque [22]. Imaging has also demonstrated a higher tendency toward tendon elongation in these patients [31].

Even so, these objective differences often fail to translate into meaningful discrepancies in patient-reported outcomes or long-term career longevity among professional athletes [17]. Patient-reported outcomes, such as the Achilles tendon Total Rupture Score (ATRS), improve steadily in both surgical and nonoperative cohorts, with no consistent differences when functional rehabilitation is employed [32]. Athletes often describe near-complete recovery, reporting reduced pain, stiffness, and strong confidence in returning to play. Yet objective testing tells a different story. Heel-rise endurance assessments and dynamometry consistently reveal persistent asymmetries, more pronounced in nonoperative patients [32].

Overall, operative repair appears to confer modest biomechanical advantages, particularly in preserving tendon length and push-off power. Yet nonoperative treatment, when paired with structured early rehabilitation, achieves equivalent long-term satisfaction, comparable ATRS gains, and avoids surgical morbidity. In practice, both pathways allow the majority of athletes to return to play, with rehabilitation quality serving as the decisive factor in functional recovery.

Discussion

The debate between operative and nonoperative management of Achilles tendon rupture has shifted from a binary choice to a patient-specific decision guided by activity level, comorbidities, and rehabilitation potential. Meta-analyses and RCTs [5,16,17] show that with early functional rehabilitation, rerupture rates and patient-reported outcomes are broadly comparable across strategies, with surgery trading a modest reduction in rerupture for more procedure-related complications. Operative repair, however, continues to offer modest biomechanical advantages in some cohorts (e.g., tendon length preservation/plantar-flexion torque), which may matter for elite, power-dependent athletes, but these potential benefits must be balanced against higher rates of wound issues and sural nerve injury seen more often with surgical approaches [19,32]. For recreational or elderly athletes, nonoperative management may therefore represent the safer and more cost-effective option, minimizing perioperative morbidity while achieving similar long-term function and quality of life [33]. 

Beyond structural recovery, psychological readiness and rehabilitation quality have emerged as central determinants of successful RTP. Persistent fear of rerupture, diminished confidence, and altered movement patterns can impede performance long after tendon healing; validated tools such as the ATRS, the Tampa Scale for Kinesiophobia (TSK), and the ankle ligament reconstruction-return to sport injury (ALR-RSI) adapted/validated for Achilles repair can help quantify these factors and guide targeted interventions [34,35]. Emerging evidence on biologic adjuncts remains mixed; the highest-quality RCT of PRP for acute Achilles tendon rupture found no functional benefit, while broader syntheses show heterogeneity and inconsistent effects, supporting a cautious, study-driven approach rather than routine use [15]. Table 2 provides a summary of the findings of the most relevant studies about Achilles tendon rehabilitation.

**Table 2:** Summary of recent evidence on operative vs. nonoperative management of Achilles tendon rupture ATRS: Achilles Tendon Total Rupture Score, RCT: Randomized controlled trial, RTP: Return to play, PROs: Procedures, rehabilitation, and outcomes

Source	Primary outcomes	Study design	Findings	Level of evidence
Willits et al., 2010 [16]	Rerupture, function	Multicenter RCT	Early rehab: no rerupture difference; surgery ↑ complications	Level I
Myhrvold et al., 2022 [5]	Rerupture, function	Multicenter RCT	Larger trial: surgery 0.6% rerupture vs. nonoperative 6.2%	Level I
Khan et al., 2005 [4]	Acute rupture outcomes	Meta-analysis (RCTs)	Surgery ↓ rerupture but ↑ complications; early rehab narrowed gap	Level I
Soroceanu et al., 2012 [3]	Surgery vs. nonoperative	Meta-analysis (RCTs)	Surgery ↓ rerupture but ↑ complications; early rehab reduced differences	Level I
Ochen et al., 2019 [17]	Operative vs. nonoperative	Systematic review and meta-analysis	Functional outcomes equivalent; surgery ↑ complications	Level I
Seow et al., 2023 [25]	Complications, rerupture	Systematic review of overlapping meta-analyses	Surgery ↓ rerupture, ↑ complications	Level I
Kołodziej et al., 2013 [33]	Complications, efficacy	Prospective RCT (preliminary)	Minimally invasive, reduced wound complications; outcomes comparable	Level II
Mullaney et al., 2006 [15]	Plantarflexion strength	Cohort	Persistent end-range plantarflexion weakness after repair	Level III
Caolo et al., 2021 [16]	Outcomes, complications	Retrospective cohort	Minimally invasive repair ↓ wound issues vs. open	Level III
Hsu et al., 2015 [26]	Outcomes, complications	Retrospective cohort	Percutaneous repair ↓ wound issues vs. open; similar function	Level III
Trofa et al., 2017 [8]	RTP in pro athletes	Athlete cohort	Most pros returned, but performance declined	Level III
Nilsson-Helander et al., 2007 [34]	ATRS score validation	Validation study	ATRS reliable, valid for PROs	Level II

Limitations

The present review is limited by substantial methodological heterogeneity among included studies, varying rehab protocols (weight-bearing timelines, ROM progression), inconsistent outcome definitions (e.g., rerupture, RTP), and nonuniform strength testing and imaging methods, hindering direct cross-study comparisons [33]. Interpretation is also constrained by mixed athletic populations with few elite/pro cohorts, limited follow-up beyond two years, and variable reporting of rehab adherence/supervision and access to specialized resources; publication bias and inconsistent RTP criteria further complicate synthesis [36]. Cost-effectiveness and patient-preference data remain underreported in many comparative series, limiting shared decision-making granularity [33].

Future directions

Development and prospective validation of patient-selection algorithms, such as the CARTA, should continue, with explicit testing in elite populations and imaging-based criteria (e.g., overlap/elongation) built into pathways [6]. Standardizing rehabilitation content and progression (weight-bearing, dorsiflexion advancement, milestones to plyometrics/change-of-direction) would improve evidence synthesis and reproducibility across centers [36]. Finally, precision rehab could be advanced by integrating quantitative imaging (e.g., qMRI/ultrasound metrics of tendon quality and elongation) and wearable load-monitoring to capture real-world tendon loading during boot ambulation and return-to-sport transitions [37-39].

## Conclusions

Achilles tendon rupture is a complex injury requiring individualized management rather than a one-size-fits-all approach. Modern functional rehabilitation has minimized differences in rerupture rates and outcomes between surgical and non-surgical care. While operative repair may benefit elite athletes seeking maximal strength, non-operative treatment offers similar functional recovery with fewer complications. Ultimately, optimal outcomes depend less on the surgical choice and more on structured rehabilitation, psychological readiness, and patient-centered care.
